# Percutaneous Transluminal Angioplasty in Patients with Peripheral Arterial Disease Does Not Affect Circulating Monocyte Subpopulations

**DOI:** 10.1155/2016/2708957

**Published:** 2016-10-13

**Authors:** Pawel Maga, Tomasz P. Mikolajczyk, Lukasz Partyka, Marek Krzanowski, Krzysztof P. Malinowski, Rafal Nizankowski

**Affiliations:** ^1^Department of Angiology, Jagiellonian University Medical College, Kraków, Poland; ^2^ANGIO-MEDICUS Angiology Clinic, Kraków, Poland; ^3^Department of Internal and Agricultural Medicine, Jagiellonian University Medical College, Kraków, Poland; ^4^Institute of Cardiovascular and Medical Sciences, University of Glasgow, Glasgow, UK; ^5^Faculty of Health Sciences, Jagiellonian University Medical College, Kraków, Poland

## Abstract

Monocytes are mononuclear cells characterized by distinct morphology and expression of CD14 and CD16 surface receptors. Classical, quiescent monocytes are positive for CD14 (lipopolysaccharide receptor) but do not express Fc gamma receptor III (CD16). Intermediate monocytes coexpress CD16 and CD14. Nonclassical monocytes with low expression of CD14 represent mature macrophage-like monocytes. Monocyte behavior in peripheral arterial disease (PAD) and during vessel wall directed treatment is not well defined. This observation study aimed at monitoring of acute changes in monocyte subpopulations during percutaneous transluminal angioplasty (PTA) in PAD patients. Patients with Rutherford 3 and 4 PAD with no signs of inflammatory process underwent PTA of iliac, femoral, or popliteal segments. Flow cytometry for CD14, CD16, HLA-DR, CD11b, CD11c, and CD45RA antigens allowed characterization of monocyte subpopulations in blood sampled before and after PTA (direct angioplasty catheter sampling). Patients were clinically followed up for 12 months. All 61 enrolled patients completed 12-month follow-up. Target vessel failure occurred in 12 patients. While absolute counts of monocyte were significantly lower after PTA, only subtle monocyte activation after PTA (CD45RA and *β*-integrins) occurred. None of the monocyte parameters correlated with long-term adverse clinical outcome. Changes in absolute monocyte counts and subtle changes towards an activation phenotype after PTA may reflect local cell adhesion phenomenon in patients with Rutherford 3 or 4 peripheral arterial disease.

## 1. Introduction

Monocytes account for 3% to 8% of peripheral blood leukocytes. They are mononuclear cells characterized by specific morphology but more accurately by their expression of CD14 and CD16 surface receptors [1]. They represent the main component of the innate immunity, and their default mode of action is phagocytosis. However, they are also involved in endogenous inflammatory processes including atherosclerotic plaque formation. Monocytes promote leukocyte recruitment to plaques and activate inflammatory signaling pathways, such as nuclear factor kappa-B. Monocytes [2] and cholesterol-loaded macrophages are a characteristic histology finding of atherosclerotic plaque and seem to be major players in all stages of atherosclerotic plaque development. This process starts with monocyte adhesion to endothelial cells mediated by selectins and integrin ligands. Subsequent rolling of monocytes on activated endothelial cells is dependent on the interaction of E-selectin with monocytic P-selectin glycoprotein ligand-1 (PSGL-1) [3]. Firm attachment between cells is possible as a result of interaction between endothelial integrin ligands, such as VCAM-1 (vascular cell adhesion molecule-1) and ICAM-1 (intercellular adhesion molecule-1) and monocytic VLA-4 (very late antigen-4) or LFA-1 (lymphocyte function-associated antigen 1) and MAC-1 (macrophage-1 antigen) [4]. After adhesion, monocytes migrate to the intimal layer of vascular wall, the process that is PECAM-1 (platelet-endothelial cell adhesion molecule-1, CD31) and CD99 dependent [5].

The extent of the inflammatory infiltrates and their strategic location within the protective fibrous cap is associated with plaque rupture and thrombosis [6]. Adventitial inflammation has also been described and is linked with an expansion of the adventitial* vasa vasorum* in unstable atherosclerosis [7]. Macrophage differentiation from monocytes is critical phenomenon for the development of atherosclerosis [8]. Macrophages are heterogeneous cells, and when they are appropriately activated, they phagocytose cytotoxic lipoproteins, clear apoptotic bodies, secrete anti-inflammatory cytokines, and synthesize matrix repair proteins that stabilize vulnerable plaques [9]. Morphology studies show the histological similarity of atherosclerotic plaque initiation and development independent of actual lesion location [10].

Three key populations of monocytes in humans were characterized (see Table 1).

Classical, quiescent monocytes are positive for CD14 (lipopolysaccharide (LPS) receptor) but do not express Fc gamma receptor III (CD16). CD16+ monocytes coexpressing CD14 possess proinflammatory and proatherogenic properties, although their nature varies depending on the level of CD14 expression. Nonclassical cells with low expression of CD14, that is, CD14+CD16++, represent a mature macrophage-like monocyte, being an important source of tumor necrosis factor-alpha (TNF-alpha), while the role of intermediate CD14++CD16+ monocytes is less clear. Both intermediate and nonclassical monocyte populations are linked to the pathogenesis of the cardiovascular disease [12, 13] and with myocardial dysfunction and recovery following myocardial infarction, although their relationship to subclinical atherosclerosis is less clearly defined [14].

Both clinical and experimental studies suggest that classical and intermediate populations play a role in poor short-term and intermediate-term cardiovascular outcomes.

Still, monocyte biology in atherogenesis is poorly understood, and puzzling findings remain. No coherent association between monocyte counts and cardiovascular (CV) disease was observed in large epidemiological studies [15, 16]. CD14++CD16+ monocytes independently predicted cardiovascular events in subjects referred for elective coronary angiography [17]. Also, CD14++CD16+ monocytes but not total monocyte numbers were independently associated with CV events in both nondialysis [18] and dialyzed [13] chronic kidney disease patients.

Peripheral arterial disease (PAD) is a vascular disorder of atherosclerotic origin [10]. Lower extremity peripheral artery disease is the third leading cause of atherosclerotic cardiovascular morbidity, following coronary artery disease and stroke. PAD is a global disease with exponential growth also in developing countries [19]. By 2020, cardiovascular diseases are predicted to be the major causes of morbidity and mortality in most developing nations around the world [20].

It is not clear whether endovascular treatment of PAD allows for postponing and avoiding deleterious effects of peripheral ischemia such as critical limb ischemia (CLI) and major amputation. Patients with CLI are at particular risk of major cardiac and cerebrovascular events and over 20% of them die within a year after CLI diagnosis [21].

Previous studies reported the correlation of systemic inflammatory status and progression of peripheral arterial disease (PAD) [22]. Several inflammatory biomarkers, including high sensitivity C-reactive protein (CRP) [23], soluble adhesion molecules (such as sICAM [24]), and tumor necrosis factor-*α* (TNF-*α*) [25], are associated with both the incidence and severity of PAD [26]. Inflammation increases the risk of adverse graft outcomes in PAD patients who have undergone peripheral bypass surgery [27], and greater systemic inflammation is associated with increased cardiovascular morbidity and mortality in individuals with PAD [28]. Monocytes were the only WBC type significantly and independently associated with PAD in a representative sample of the US population after adjustment for other inflammatory markers [29]. Additionally, monocytes from PAD patients were shown to form aggregates with platelets* in vivo* [30] and adhere easier to cultured endothelial cells [31].

Endovascular treatment of PAD allowed controlling symptomatic disease in many cases. It is minimally invasive and is still a very efficient way to combat arterial stenosis and occlusion. Acute changes in circulating cell populations were demonstrated in course of endovascular treatment [32]. Moreover, endovascular procedure is a unique opportunity to access and evaluate direct vicinity of atherosclerotic plaque* in vivo*.

Both plaque contents and subendothelial space are exposed during percutaneous transluminal angioplasty (PTA). It was recently demonstrated [33] that subendothelial resistin activated smooth muscle cells (SMC) by upregulating fractalkine, MCP-1 expression, and monocyte chemotaxis. Except for activation of Toll-like receptor 4 (TLR4) and Gi/o proteins, both fractalkine and MCP-1 enhanced recruitment of monocytes to subendothelial space involving CX3CR1 and CCR2 receptors.

Therefore, we hypothesized that acute changes in monocyte subpopulations may occur during PTA. And this study aimed at evaluation of monocyte subpopulations in PAD patients during the peripheral transluminal angioplasty (PTA) procedure.

## 2. Materials and Methods

### 2.1. Setting

Patients were recruited at the tertiary angiology center at the university hospital and the largest provider of peripheral arterial endovascular therapy in the Malopolska region of Poland, serving a population of approximately 3.3 million inhabitants. The treatment details of patients admitted to the department were entered prospectively into a specific database called the Malopolska Endovascular Registry [34, 35].

### 2.2. Patients

Patients with PAD who required endovascular treatment for severe claudication or rest pain and met all inclusion criteria entered the study group.

Patients with any signs of inflammation, ischemic and trophic ulcerations, chronic kidney disease, neoplastic disease, or elevated CRP were excluded. Body temperature was measured on the day preceding procedure and immediately before the procedure. They were consequently evaluated for symptoms and signs of peripheral ischemia (including baseline Rutherford's scale classification and ankle-brachial index (ABI)). Preprocedural imaging studies (ultrasound color Doppler or CT angiography) helped in the proper planning of endovascular treatment. PTA was performed in iliac, femoral, or popliteal vascular segments.

Thus, the group studied corresponded to stage 3 or stage 4 of PAD using Rutherford's classification. All subjects in this study received an identical treament consisting of antiplatelet aspirin (75 mg) as well as atorvastatin (20–40 mg) and clopidogrel (75 mg). All patients signed an informed consent form and the local Jagiellonian University Ethical Committee accepted this study (KBET/282/B/2013). Due to the observational character, the study was not submitted to the public trial database.

### 2.3. Procedure and Blood Sampling

Vascular access was achieved in all cases by common femoral artery puncture and 6F arterial introducer. After assuring arterial access, a diagnostic angiography of target vessel was performed with administration of ca. 30 mL of Ultravist 370 contrast medium (Ultravist® (iopromide) Injection contrast medium, Bayer, Germany). Bolus injection of 5000 IU of unfractionated heparin was given after diagnostic angiography in all patients. Attention was paid to avoid any modifications of preparation or treatment protocol to minimize confounders.

Sample 0 was collected after diagnostic angiography directly from the balloon catheter. Balloon catheters were sized to reference vessel diameter with quantitative angiography. Sample 1 was collected from balloon catheter at the level of balloon deflation (direct vicinity of the target atherosclerotic plaque) immediately after balloon deflation (lasting 30 to 60 seconds). The time between two samplings did not exceed 15 minutes. Blood (10 mL) was sampled to ethylenediaminetetraacetic acid- (EDTA-) containing vials, immediately placed on ice, transferred to the laboratory within 45 minutes, and analyzed.

### 2.4. Patients' Follow-Up

All patients were monitored for clinical results until month 12. Symptomatic follow-up, ABI measurement, and ultrasound were used to verify vessel reocclusion.

### 2.5. Flow Cytometry Analysis of Antigen Expression on Peripheral Blood Monocytes

Surface antigens were studied in peripheral blood mononuclear cells (PBMCs) as previously described [36]. Briefly, PBMCs were isolated by gradient centrifugation using Pancoll human (PAN-Biotech GmbH, Aidenbach, Germany) from the ethylenediaminetetraacetic acid- (EDTA-) treated blood. PBMCs were further suspended in phosphate-buffered saline (PBS) containing 1% heat-inactivated fetal bovine serum (FBS) (Gibco, Life Technologies, USA) and were used immediately after isolation. A total of 500,000 PBMCs were stained for 20 minutes with fluorochrome-conjugated monoclonal antibodies: anti-CD14-APC-H7 (clone MΦP9), anti-CD16-PE (clone 3G8), anti-human leukocyte antigen (HLA)-DR-PE-Cy7 (L243), anti-CD11b/Mac-1-Pacific Blue (clone ICRF44), anti-CD11c-APC (clone B-LY6), and anti-CD45RA-FITC (clone L48) (BD, Pharmingen, CA, USA). After staining, cells were washed twice with PBS containing 1% FBS. Cells were processed in the FACS Verse flow cytometer and analyzed using FlowJo software (Tree Star, USA). Monocytes were gated according to forward scatter (FSC) and side scatter (SSC) signals as described previously. Subsequently, cells were gated in an HLA-DR/CD14 plot to exclude HLA-DR-negative Natural Killer cells. Finally, we analyzed cells for CD14 and CD16 expression, which allowed for discrimination of major monocyte subpopulations: CD14++CD16−, CD14++CD16+, and CD14+CD16++.

### 2.6. Statistics

Data were described using mean with standard deviations or median with the first and third quartiles for continuous variables and as counts and percentages for nominal variables. Continuous variables were compared using Students *t*-test with or without correction for unequal variances (verified using Levene's test) or Mann–Whitney *U* test for not normally distributed data (verified using Shapiro-Wilk test). Data before and after the intervention were compared using paired version of tests above. Relative and absolute changes were also analyzed. Nominal variables were compared using Pearson's *χ* ^2^ test or Fisher's exact test when appropriate. Logistic regression and Cox's proportional-hazards models were calculated to analyze the relationship of total monocyte and monocyte subset cell counts with event-free survival after adjustment for age and sex to determine independent predictors of reintervention.

Data management, statistical analysis, and statistical discovery were performed using JMP®, version 12., SAS Institute Inc., Cary, NC, 1989–2012, and the software environment for statistical computing, R. Two-sided *p* values < 0.05 were considered significant.

## 3. Results

A group of 61 patients with PAD who required PTA were enrolled in the study. It was a small group but reflected a usual profile of patients with a high burden of cardiovascular risk. General patient characteristics are given in Table 2.

Patients were scheduled to receive endovascular treatment in either the iliac, femoral, or popliteal arteries at our department. The key clinical criterion for inclusion was severe claudication or resting pain. Ischemia was evaluated by Rutherford's scale, ankle-brachial index (ABI), and examination of arteries using USG with color Doppler or CT angiography. Ischemic symptoms evaluated by Rutherford's scale equaled 3 in 50 subjects and 4 in 11 others. In 21 cases (34.4%), only PTA was performed, whereas 40 cases (65.6%) required PTA with stent implantation. However, samples were taken exclusively before and after angioplasty. No previous revascularization was performed in the target vessel. No signs of the inflammation were present in all cases at the time of PTA procedure. Body temperature was below 37°C, and peripheral blood leukocytes were within reference range.

During 12-month observation period, 12 cases of clinical and/or imaging target vessel failure were observed. No patients were lost to follow-up. There were no cases of major cardiac and cerebrovascular events such as cardiovascular death, acute myocardial infarction, or nonhemorrhagic stroke in the studied group during the observation period.

Absolute amounts of monocytes isolated before PTA were higher than those after PTA (621 ± 159 cells/mm^3^ versus 556 ± 146 cells/mm^3^, *p* = 0.028, Sample 0 versus Sample 1, resp.). However, no significant differences were observed between the relative numbers of isolated PBMCs before and after PTA.

**Figure 1:**
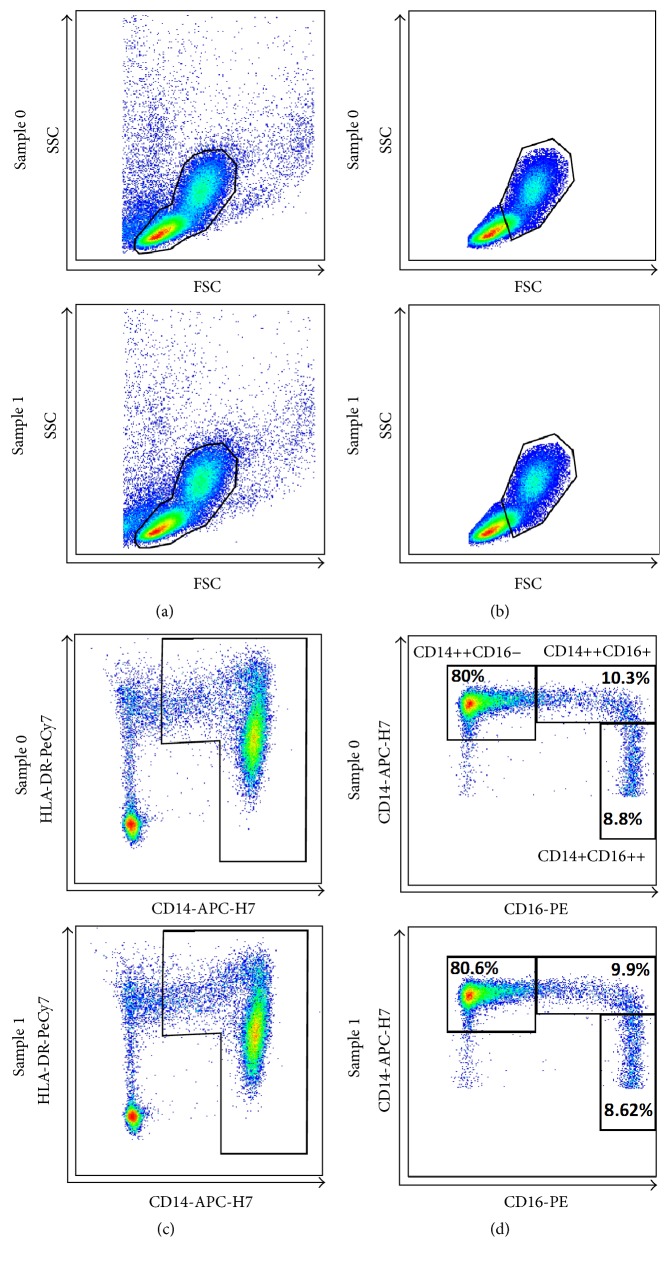
Flow cytometric analysis of monocytes and their subsets from PBMC. Monocytes with a part of lymphocytes (b) were gated according to forward scatter (FSC) and side scatter (SSC) from peripheral blood mononuclear cells (PBMCs (a)). Subsequently, cells were gated in an HLA-DR/CD14 plot to exclude HLA-DR-negative Natural Killer cells (c). Finally, monocyte subpopulations were identified according to CD14 and CD16 expressions (CD14++CD16−, CD14++CD16+, and CD14+CD16++ (d)). Percentages of major monocyte subpopulations are indicated (d) in Sample 0 (before PTA) and Sample 1 (after PTA).

**Figure 2:**
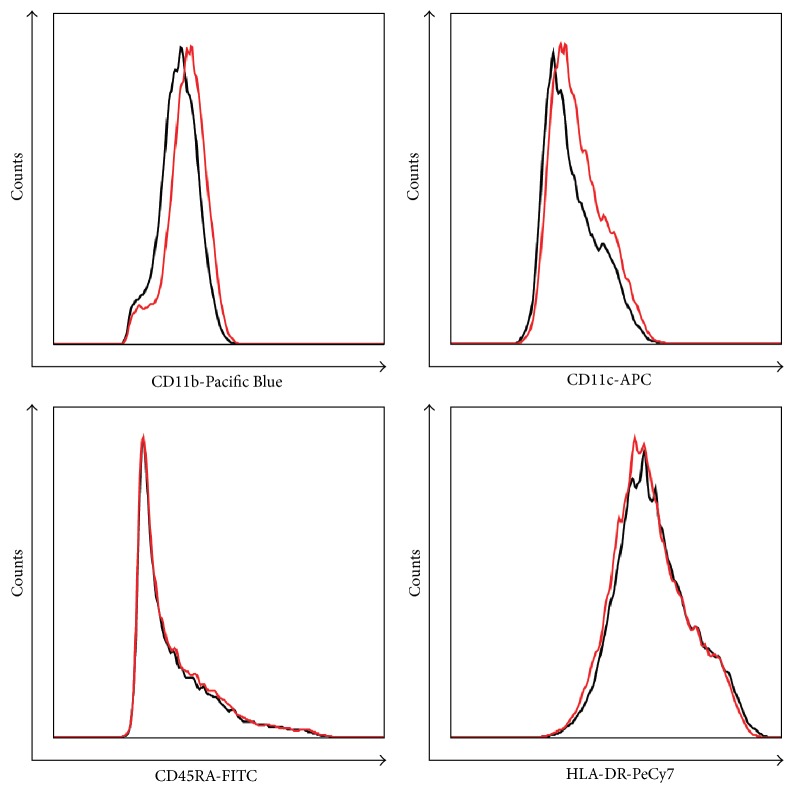
Examples of antigen expression on peripheral blood monocytes. Surface antigen expressions of CD11b, CD11c, CD45RA, and HLA-DR were studied on peripheral blood monocytes using flow cytometry. Black histograms indicated antigen expression on cells isolated from the sample before PTA. Red histogram indicated antigen expression after PTA.

Flow cytometry analysis was performed, and representative pictures are presented in Figures 1 and 2.

No differences were found in monocyte subpopulations before and after PTA (Table 3).

Significant but subtle changes were observed in mean fluorescence of CD11b both in total monocyte population (997 ± 362 versus 1071 ± 383, *p* = 0.04) and in CD14++CD16− (1029 ± 387 versus 1113 ± 419, *p* = 0.04) and CD14++CD16+ (1255 ± 458 versus 1323 ± 461, *p* = 0.045) subsets before and after PTA.

Also, mean fluorescence of CD11c (703 ± 209 versus 775 ± 260, *p* = 0.03) and CD45RA (1779 ± 412 versus 1808 ± 414, *p* = 0.03) in CD14++CD16− population was significantly higher after PTA.

Attempts were made to compare and find independent predictors of worse treatment outcome (target vessel failure at 12 months). However, none of the investigated monocyte-related markers was significant in this respect, also after adjustment for age and gender. It concerned both absolute values of pre-PTA and post-PTA samples and differences between them.

No systematic difference was noted between patients with Rutherford classes 3 and 4. Also, stent implantation was not a significant confounder in this clinical setting.

## 4. Discussion

PAD is considered as a strong risk factor for the premature development of atherosclerosis and cardiovascular events. The mechanisms of this increased risk remain unclear, although there were some previous reports linking immune mechanisms and monocyte participation particularly in this process.

Leukocyte activation was also suggested to be one of the major factors of detrimental short-term and mid-term treatment results after coronary angioplasty [37, 38]. Additionally, monocyte chemoattractant protein-1 (MCP-1) was clearly linked to adverse outcomes of endovascular treatment both in coronary circulation and in peripheral circulation [39].

We have observed reduction in total monocyte count during PTA and subtle changes towards activation pattern in monocyte subpopulations. They did not correlate with target vessel failure in long-term follow-up. This is essentially matching recent findings of DeSart et al. [40]. They demonstrated that endovascular revascularization had only modest influence on the overall activation state of the systemic inflammatory system, with baseline variability exceeding the perturbations induced by the intervention. Conversely, changes in the monocyte genome are evident during 28 days, promoting leukocyte extravasation. Additionally, no single plasma protein predictor correlated with outcome, but a pattern of genes regulating cell-cycle signaling showing up after the endovascular procedure was predictive of clinical outcome.

CD45RA is considered as a marker of activation of peripheral blood monocytes [41] and activation of monocytes* in vitro* induces their CD45RA expression [42].

The *β*-integrins (CD11b/CD18, CD11c/CD18) expressed on peripheral blood monocytes can interact with their ligands including intercellular adhesion molecule-1 (ICAM-1) and vascular cell adhesion molecule-1 (VCAM-1) on activated endothelium [43, 44].

An approach to evaluate blood sampled locally from the vicinity of recently treated atherosclerotic plaque does not seem to be substantiated at least in acute results of angioplasty on the expression pattern of surface markers of monocyte subsets. Lower total amounts of monocytes after PTA suggest that they are not released from atherosclerotic plaque, but they may adhere to the vessel wall denuded of endothelium during the angioplasty treatment.

Subtle differences in CD45RA, CD11b, and CD11c expressions were particularly seen in selected monocyte subsets. This may reflect migration of cells into the subendothelial space, adhesion of cells in sites of endothelial injury, or release of cytokines, resulting in acute subpopulation shift.

The study also had some inherent limitations. The natural follow-up period was 12 months, and it was treatment related and not event related, as in previous studies. Although a possible influence of heparin or contrast media administration for surface expression of antigens exists, all patients received identical treatment in order to minimize this possible bias.

There was no external disease-free control group. However, previous reports exist on the differences in peripheral monocyte population between healthy subjects, claudicants, and critical limb ischemia patients [45]. In this study, we were interested in acute changes in monocyte population during an endovascular treatment.

While it would be very valuable to evaluate other monocyte activation methods such as concentration of activation-specific human anti-alphaMbeta2 single-chain antibodies [46] or expression and release of chemokines by transcriptome and chemokinome profiling [47], we were not able to perform monocyte-specific stimulation studies at the same time due to grant limitations.

It was an observational cohort study and no causality could be determined, but further investigation of possible mechanisms is continued and seems mandated.

## 5. Conclusion

Peripheral percutaneous transluminal angioplasty induced reduction in total monocyte count in samples from direct vicinity of target lesion. No significant acute changes in the composition of monocyte subpopulation in patients with Rutherford 3 or 4 peripheral arterial disease were noted, except for subtle shift towards an activation pattern (CD45RA and *β*-integrins, CD11b and CD11c). A sampling of leukocytes from angioplasty catheter (i.e., the vicinity of target atherosclerotic plaque) provides additional insight into the local environment of nonspecific immunological response even in acute observation.

## Figures and Tables

**Table 1:** Differential expression patterns of crucial markers of key monocyte subsets [11].

Subset	Markers
CD14++CD16− (classical)	CCR2, FcgRI, CD62L, PSGL-1, IL-6R, SR-A, CD36
CD14++CD16+ (intermediate)	CCR5, CD163, TLR4, HLA-DR, CD105, Tie2, VEGFR1/2
CD14+CD16++ (nonclassical)	CX3CR1, CD49d, sialophorin, SLAN

**Table 2:** Demographic and clinical data of 61 patients undergoing monocyte population sampling and evaluation.

Variable	*n* (%) or value
Age (mean ± SD; median)	62.2 ± 10.8; 61 years
Male gender	50 (82%)
Hypertension	33 (54%)
Diabetes	
Type 2	16 (26%)
Ischemic heart disease	25 (41%)
Impaired renal function (GFR < 70 mL/min/1.73 m^2^)	0 (0%)
Dialysis dependence	0 (0%)
Smoking	
Prior	40 (66%)
Current	16 (26%)
Hyperlipidaemia	30 (49%)
Congestive heart failure	0 (0%)
Rutherford-Becker class	
3	50 (82%)
4	11 (18%)
Reocclusion within 6-month observation period	12 (20%)

**Table 3:** Characteristics of monocyte subsets defined by expression of CD14 and CD16 in samples from PAD patients before and after PTA.

	CD14++CD16− (classical) %	CD14++CD16+ (intermediate) %	CD14+CD16++ (nonclassical) %
Before PTA (Sample 0)	76.9 ± 7.9	10.2 ± 3.7	10.5 ± 4.9
After PTA (Sample 1)	77.7 ± 7.9	9.9 ± 4.3	10.0 ± 4.6
